# Serum iron is closely associated with metabolic dysfunction-associated fatty liver disease in type 2 diabetes: A real-world study

**DOI:** 10.3389/fendo.2022.942412

**Published:** 2022-09-05

**Authors:** Jun-Wei Wang, Chun-Hua Jin, Jiang-Feng Ke, Yi-Lin Ma, Yu-Jie Wang, Jun-Xi Lu, Mei-Fang Li, Lian-Xi Li

**Affiliations:** ^1^ 1Department of Endocrinology and Metabolism, Shanghai Sixth People’s Hospital Affiliated to Shanghai Jiao Tong University School of Medicine, Shanghai Clinical Center for Diabetes, Shanghai Diabetes Institute, Shanghai Key Laboratory of Diabetes Mellitus, Shanghai Key Clinical Center for Metabolic Disease, Shanghai, China; ^2^ Department of Endocrinology and Metabolism, Shanghai Songjiang District Central Hospital, Songjiang Hospital Affiliated to Shanghai Jiaotong University School of Medicine (Preparatory Stage), Shanghai, China; ^3^ Department of Emergency, Shanghai Sixth People’s Hospital Affiliated to Shanghai Jiao Tong University School of Medicine, Shanghai, China

**Keywords:** serum iron, non-alcoholic fatty liver disease, metabolic dysfunction-associated fatty liver disease, type 2 diabetes, insulin resistance

## Abstract

**Aims:**

There is still a debate about the relationship between serum iron and metabolic dysfunction-associated fatty liver disease (MAFLD). Furthermore, few relevant studies were conducted in type 2 diabetes mellitus (T2DM). Therefore, this study aimed to explore the association of serum iron levels with MAFLD in Chinese patients with T2DM.

**Methods:**

This cross-sectional, real-world study consisted of 1,467 Chinese T2DM patients. MAFLD was diagnosed by abdominal ultrasonography. Based on serum iron quartiles, the patients were classified into four groups. Clinical characteristics were compared among the four groups, and binary logistic analyses were used to assess the associations of serum iron levels and quartiles with the presence of MAFLD in T2DM.

**Results:**

After adjusting for gender, age, and diabetes duration, significantly higher prevalence of MAFLD was found in the second (45.7%), third (45.2%), and fourth (47.0%) serum iron quartiles than in the first quartiles (26.8%), with the highest MAFLD prevalence in the fourth quartile (*p* < 0.001 for trend). Moreover, increased HOMA2-IR (*p* = 0.003 for trend) and decreased HOMA2-S (*p* = 0.003 for trend) were observed across the serum iron quartiles. Fully adjusted binary logistic regression analyses indicated that both increased serum iron levels (OR: 1.725, 95% CI: 1.427 to 2.085, *p* < 0.001) and quartiles (*p* < 0.001 for trend) were still closely associated with the presence of MAFLD in T2DM patients even after controlling for multiple confounding factors.

**Conclusions:**

There is a positive correlation between the presence of MAFLD and serum iron levels in T2DM patients, which may be attributed to the close association between serum iron and insulin resistance. Serum iron levels may act as one of the indicators for evaluating the risk of MAFLD in T2DM individuals.

## Introduction

In addition to oxygen transport, iron also has a vital role in many metabolic processes with the potential to cause oxidative damage when in excess (1). Specifically, serum ferric iron is carried by transferrin and transported into the cell, where it is reduced to ferrous iron (2). Ferrous iron facilitates peroxidation of membrane-bound, PUFA-containing lipids and triggers propagation of lipid peroxidation, which cause damage to mitochondria and other organelles and finally lead to the development and progression of metabolic disorders (2). Therefore, iron overload may be linked to multiple metabolic disorders such as obesity, hyperlipidemia, hyperglycemia, and insulin resistance (1, 3, 4). For example, a recent study indicated increased serum iron levels in patients with type 2 diabetes mellitus (T2DM) (4). Moreover, the risk of developing diabetes induced by iron was probably close to the relative risk generated by obesity (1). Additionally, it was also noted that iron metabolism disorders were remarkably correlated with insulin resistance and obesity (3).

As one of the metabolic disorders, metabolic dysfunction-associated fatty liver disease (MAFLD), formerly named non-alcoholic fatty liver disease (NAFLD), is referred to as the “hepatic manifestation of the metabolic syndrome” and causes a progressive liver disease group including steatosis, fibrosis, cirrhosis, and hepatocellular carcinoma (5). MAFLD is centered on hepatic fat accumulation and comorbid with obesity, T2DM, or evidence of metabolic dysregulation, with a prevalence of 25.9%–38.0% in the general population (6). Moreover, a recent meta-analysis estimated that T2DM patients had a higher risk developing MAFLD than the general population, with a prevalence of 55.5%–70% (7, 8). In our recent studies, the prevalence of MAFLD was 39.4%–52.6% in patients with T2DM, which was also higher than that in the general population (9–11). Given the high prevalence of MAFLD in T2DM, early identification and intervention of risk factors associated with MAFLD will be beneficial in reducing the occurrence of MAFLD in T2DM subjects.

Currently, some studies have identified several iron-related serum markers such as serum ferritin, hepcidin, and serum transferrin saturation, which are closely associated with MAFLD (12–14). However, the relationship between serum iron and MAFLD is infrequently studied and probably not explicit in general and diabetic populations. For example, in a small sample study of mostly biopsy-proven NAFLD, only two (3%) patients had high serum iron levels (15). Additionally, a negative correlation between serum iron and NAFLD prevalence in the general population was found, as the U.S. National Health and Nutrition Examination Survey (NHANES) data indicated (16). However, several studies pointed to the presence of unchanged serum iron levels in patients with NAFLD (17–19). Moreover, a previous study emphasized an elevated serum iron level in NAFLD patients in comparison to patients without NAFLD (20).

Notably, the relationship between iron status and MAFLD remained controversial in T2DM subjects, as there were few studies and clinical trials. A study including T2DM subjects highlighted that the T2DM prevalence increased in the NAFLD group compared with the non-NAFLD group, but without an increase in serum iron (21). Likewise, in a study containing nearly half of NAFLD patients with T2DM, histological iron in liver was not associated with NAFLD severity (22). However, a previous study including patients with T2DM endorsed an increase in the NAFLD prevalence with elevated serum iron levels (23).

Therefore, our aim was to investigate the correlation between serum iron levels and MAFLD diagnosed by abdominal ultrasonography in Chinese patients with T2DM.

## Materials and methods

### Subjects and study design

This cross-sectional, real-world study included T2DM patients hospitalized in the Department of Endocrinology and Metabolism, the Sixth People’s Hospital of Shanghai Jiao Tong University from January 2006 to December 2012, and some of the patient data were from our recent studies (11, 24–26). The hospital ethical review committee approved this study [approved number: 2018-KY-018(K)], with written consent obtained from all participants. Inclusion criteria incorporated T2DM diagnosed in accordance with the WHO criteria, age ≥ 17 years old, complete clinical information and biochemical parameters, and available abdominal ultrasound findings (25). After excluding the patients with disorders related to iron metabolism such as hemochromatosis, iron-deficiency anemia, menstruation within a week, and blood transfusion or donation recently; those with liver diseases caused by drugs, viral hepatitis, and other reasons excluding alcohol; and those with other serious systemic diseases or infectious diseases, 1,467 patients were classified into four groups according to the serum iron quartiles.

### Physical examination and laboratory tests

The following data were collected at admission as previously described: hypertension history, diabetes duration (DD), alcohol intake, smoking habits, use of lipid-lowering drugs (LLDs), metformin, insulin sensitizers, insulin or insulin analogs (IIAs), and physical data including height, waist and hip circumference, weight, and blood pressure (10, 11). Specifically, the definitions of hypertension, obesity, smoking, and alcohol status were described in our previous studies (11, 25).

After fasting overnight and 2 h after breakfast on the second day of admission, blood samples were collected. Serum alanine transaminase (ALT) was measured by an enzymatic rate method with the definition of elevated ALT more than 65 U/L according to our previous study (10). Serum iron was determined using colorimetric assay by a LAbOSPECT 008AS automatic biochemical analyzer (Hitachi, Japan) (27), and serum ferritin level was measured using chemiluminescence immunoassay by a cobas e 602 module (Roche Diagnostics, Germany) (28). Other laboratory parameters such as blood glucose, lipids, insulin, C-peptide, kidney function, and urine tests were measured as described previously (10, 11, 25). The homeostasis model assessment of insulin resistance (HOMA2-IR) and the homeostasis model assessment of sensitivity (HOMA2-S) were estimated using HOMA2 Calculator version 2.2.3 (11). The estimated glomerular filtration rate (eGFR) was calculated according to the formula recommended for the Chinese population [175×(serum creatinine)^−1.234^ × (age)^−0.179^(×0.79, if female)] (25).

### Abdominal ultrasonography and diagnostic criteria

The hepatic ultrasound examinations and diagnosis of hepatic steatosis were in accordance with our previous studies (10, 11). Since T2DM patients were selected as the target population in the present study, MAFLD was diagnosed as ultrasonographically verified hepatic steatosis in addition to the presence of T2DM, which was proposed by an international expert panel from 22 countries (29).

### Statistical analysis

Data were analyzed using SPSS 15.0 (SPSS Inc., Chicago, IL, USA). Normality was assessed for continuous variables and then expressed as mean ± standard deviation or median and interquartile range. In particular, the differences between the two groups were evaluated by the *t*-test or the Mann–Whitney *U* test, while the differences between multiple groups were assessed using one-way ANOVA or the Kruskal–Wallis *H* test. Chi-square tests were used to analyze categorical variables. When gender and/or age was considered as confounders, categorical variables were corrected with logistic regression, and continuous variables were adjusted with univariate linear regression models. After non-normally distributed variables were transformed by normal score transformation, binary logistic regression was done for assessing the correlation of serum iron levels and quartiles with the MAFLD presence. Five models were constructed to evaluate the association of serum iron with MAFLD. Statistical significance was set at *p* < 0.05.

## Results

### Characteristics of the study subjects

This study consisted of 1,467 inpatients with T2DM. In accordance with the serum iron quartiles with cutoffs of <10.6, 10.6–14.0, 14.1–18.0, and >18.0 μmol/L, they were classified into four groups. The subjects’ baseline characteristics grouped by serum iron quartiles are highlighted in **Table 1**. There was a significant age and sex difference among the four groups. After adjusting for sex and age, with ascending serum iron levels, IIAs usage, DD, and levels of urinary albumin excretion (UAE) and C-reactive protein (CRP) were obviously decreased, and levels of fasting plasma glucose (FPG), fasting C-peptide (FCP), 2-h postprandial C-peptide (2h C-P), low-density lipoprotein cholesterol (LDL-C), ALT, and eGFR were significantly increased (all *p* < 0.05). Additionally, there were obvious differences in LLD usage, waist circumference (WC), BMI, and values of SBP, DBP, 2-h postprandial plasma glucose (2-h PPG), creatine (Cr), total cholesterol (TC), total triglycerides (TG), high-density lipoprotein cholesterol (HDL-C), and serum uric acid (SUA) among the serum iron quartiles in T2DM patients (all *p* < 0.05). However, no obvious difference was found in hypertension and obesity prevalence, smoking status, alcohol intake, metformin and insulin sensitizers use, WHR, SBP, DBP, and glycated hemoglobin A1c (HbA1C) among the serum iron quartile groups.

**Table 1:** Characteristics of the subjects according to serum iron levels.

Variables	Q1 (*n* = 369)	Q2 (*n* = 363)	Q3 (*n* = 368)	Q4 (*n* = 367)	*p*-value	*p-value
Serum iron (μmol/L)	<10.6	10.6–14.0	14.1–18.0	>18.0	—	—
Male (*n*, %)	145 (39.3%)	158 (43.5%)	173 (47.0%)	251 (68.4%)	<0.001	<0.001
Age (years)	63 ± 13	61 ± 12	61 ± 12	56 ± 13	<0.001	<0.001
*DD (months)	120 (48–156)	96 (36–168)	84 (36–132)	60 (12–120)	<0.001	0.006
Hypertension (*n*, %)	201 (54.5%)	209 (57.6%)	216 (58.7%)	187 (51.0%)	0.146	0.205
Obesity (*n*, %)	156 (42.3%)	174 (47.9%)	166 (45.1%)	177 (48.2%)	0.327	0.461
Smoking (*n*, %)	63 (17.1%)	90 (24.8%)	101 (27.4%)	139 (37.9%)	<0.001	0.146
Alcohol (*n*, %)	36 (9.8%)	54 (14.9%)	54 (14.7%)	85 (23.3%)	<0.001	0.537
IIAs (*n*, %)	289 (78.3%)	246 (67.8%)	235 (63.9%)	226 (61.6%)	<0.001	<0.001
LLD (*n*, %)	71 (19.2%)	110 (30.3%)	106 (28.8%)	92 (25.1%)	0.003	0.004
Metformin (*n*, %)	182 (49.3%)	203 (55.9%)	199 (54.1%)	194 (52.9%)	0.331	0.334
Insulin sensitizers (*n*, %)	28 (7.6%)	45 (12.4%)	46 (12.5%)	33 (9.0%)	0.065	0.077
SBP (mmHg)	134 ± 18	133 ± 19	135 ± 18	131 ± 17	0.021	0.552
DBP (mmHg)	79 ± 10	80 ± 9	81 ± 10	81 ± 9	0.133	0.466
WC (cm)	87.95 ± 9.98	89.85 ± 10.74	89.65 ± 10.55	90.08 ± 10.17	0.043	0.039
WHR	0.91 ± 0.07	0.91 ± 0.07	0.92 ± 0.06	0.92 ± 0.07	0.257	0.253
BMI (kg/m^2^)	24.34 ± 3.56	25.04 ± 3.62	24.85 ± 3.58	25.02 ± 3.61	0.033	0.028
*FPG (mmol/L)	7.40 (5.72–9.58)	7.51 (6.11–9.59)	7.83 (6.22–9.71)	8.12 (6.69–10.19)	0.001	0.001
*2-h PPG (mmol/L)	12.90 (9.42–16.34)	13.24 (9.94–16.39)	12.72 (9.70–15.99)	13.67 (10.47–17.12)	0.099	0.008
HbA1C (%)	9.06 ± 2.56	8.67 ± 2.25	8.91 ± 2.20	8.88 ± 2.15	0.164	0.091
*FCP (ng/ml)	1.60 (0.93–2.65)	1.79 (1.10–2.74)	1.89 (1.29–2.82)	1.93 (1.24–2.76)	0.006	0.002
*2h C-P (ng/ml)	3.25 (1.75–5.26)	4.17 (2.27–6.27)	4.35 (2.55–6.70)	4.57 (2.59–7.08)	<0.001	<0.001
*TG (mmol/L)	1.19 (0.88–1.74)	1.50 (1.04–2.36)	1.44 (1.00–2.13)	1.40 (0.99–2.00)	<0.001	<0.001
TC (mmol/L)	4.37 ± 1.01	4.78 ± 1.06	4.81 ± 1.15	4.76 ± 1.03	<0.001	<0.001
HDL-C (mmol/L)	1.10 ± 0.29	1.13 ± 0.30	1.16 ± 0.30	1.16 ± 0.29	0.019	<0.001
LDL-C (mmol/L)	2.70 ± 0.79	2.96 ± 0.83	3.00 ± 0.86	3.10 ± 0.91	<0.001	<0.001
*ALT (U/L)	16 (11–25)	19 (14–29)	22 (16–36)	25 (17–37)	<0.001	<0.001
*Cr (μmol/L)	68.0 (56.0–91.0)	66.0 (56.0–82.0)	66.0 (56.0–79.3)	69.0 (58.0–79.0)	0.057	<0.001
*SUA (μmol/L)	295 (240–358)	323 (262–380)	314 (262–371)	317 (265–380)	0.006	0.027
*UAE (mg/24 h)	17.95 (8.85–64.63)	12.45 (7.47–40.67)	12.30 (7.16–27.95)	11.10 (6.73–27.16)	<0.001	<0.001
*eGFR (ml/min/1.73 m^2^)	98.8 (69.8–129.1)	102.2 (85.1–127.7)	105.6 (87.0–128.4)	110.9 (95.5–131.0)	<0.001	<0.001
*CRP (mg/L)	3.12 (0.98–8.83)	1.36 (0.57–3.42)	1.13 (0.47–2.69)	0.87 (0.40–1.85)	<0.001	<0.001

Values are expressed as the mean ± SD, or median with interquartile range, or percentages.

p-value: The p-values were not adjusted for age and sex for the trend.

p*-value: The p*-values were adjusted for sex and age for the trend.

*The Kruskal–Wallis H test was applied.

### Comparisons of MAFLD prevalence and serum iron levels stratified by sex, age, and DD

**Figure 1:**
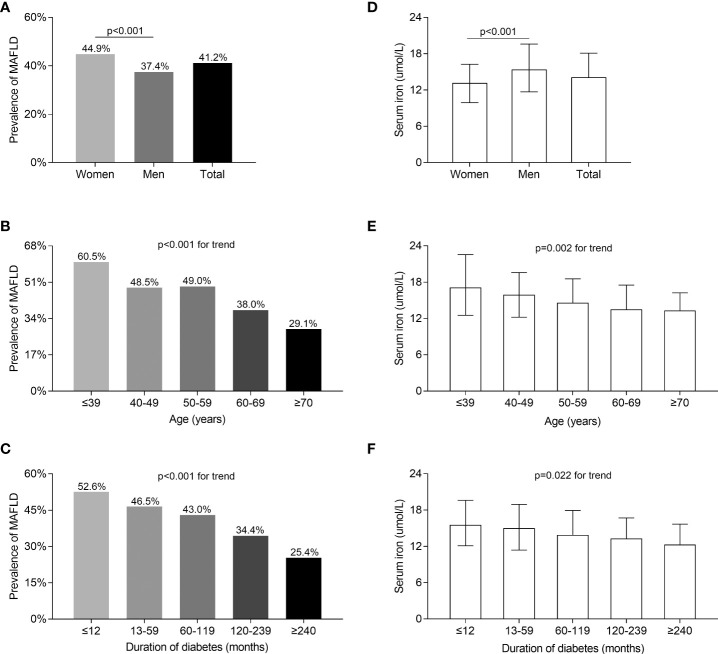
Comparisons of MAFLD prevalence and serum iron levels stratified by sex, age, and DD. **(A)** Overall prevalence of MAFLD and comparisons of the prevalence of MAFLD stratified by gender (*p* < 0.001). **(B)** Comparisons of the MAFLD prevalence among patients stratified by age (*p* < 0.001 for trend). **(C)** Comparisons of the MAFLD prevalence among patients stratified by DD (*p* < 0.001 for trend). **(D)** Overall serum iron levels and comparisons of serum iron levels stratified by gender (*p* < 0.001). **(E)** Comparisons of serum iron levels among patients stratified by age (*p* = 0.002 for trend). **(F)** Comparisons of serum iron levels among patients stratified by DD (*p* = 0.022 for trend).

**Figure 1** compares MAFLD prevalence and serum iron levels among the different gender, age, and DD groups. The MAFLD overall prevalence was 41.2%, with a higher prevalence in women (44.9%) than in men (37.4%) after adjusting for DD and age (*p* < 0.001, **Figure 1A**). However, serum iron levels were significantly lower in women than in men (*p* < 0.001, **Figure 1D**). In addition, a significant decrease in the prevalence of MAFLD was linked to increasing age (*p* < 0.001 for trend) and longer DD (*p* < 0.001 for trend) (**Figures 1B, C**). Likewise, there was a significant decline in serum iron levels with advancing age (*p* = 0.002 for trend) and prolonged DD (*p* = 0.022 for trend) (**Figures 1E, F**).

### Comparisons of serum iron levels and MAFLD prevalence

**Figure 2:**
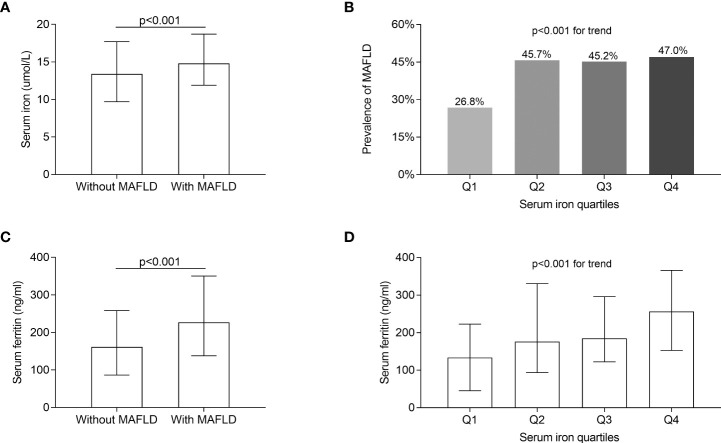
Comparisons of serum iron and ferritin levels, and MAFLD prevalence. **(A)** Comparisons of serum iron levels between T2DM patients with and without MAFLD (*p* < 0.001). **(B)** Comparisons of the prevalence of MAFLD across the serum iron quartile groups (*p* < 0.001 for trend). **(C)** Comparisons of serum ferritin between the patients with and without MAFLD (*p* < 0.001). **(D)** Comparisons of serum ferritin levels across the serum iron quartile groups (*p* < 0.001 for trend).

**Figure 2** compares the serum iron levels between T2DM patients with and without MAFLD and the prevalence of MAFLD among the serum iron quartiles. After correcting for sex, age, and DD, serum iron and ferritin levels were significantly increased in T2DM individuals with MAFLD in comparison to those without MAFLD (*p* < 0.001, **Figures 2A, C**). Moreover, significantly higher prevalence of MAFLD was found in Q2 (45.7%), Q3 (45.2%), and Q4 (47.0%) compared to Q1 (26.8%), with the highest MAFLD prevalence in Q4 after adjusting for age, sex, and DD (*p* < 0.001 for trend) (**Figure 2B**). Additionally, there was an increased trend of serum ferritin across serum iron quartiles (*p* < 0.001 for trend) (**Figure 2D**).

### Comparisons of serum ALT levels

**Figure 3:**
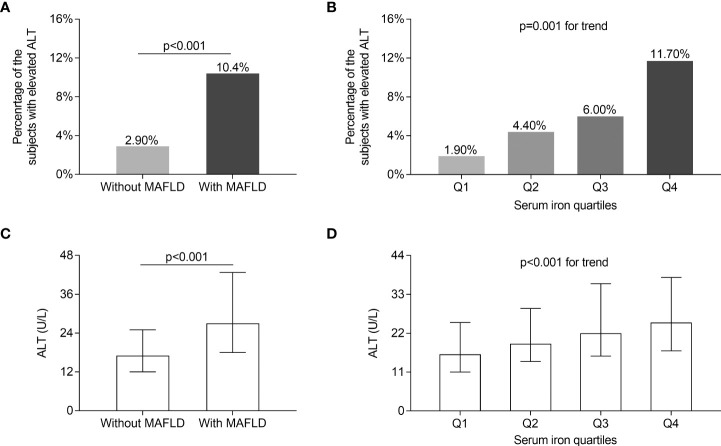
Comparisons of serum ALT levels. **(A)** Comparisons of the percentage of the subjects with elevated ALT levels between the patients with and without MAFLD (*p* < 0.001). **(B)** Comparisons of the percentage of the subjects with elevated ALT levels across the serum iron quartile groups (*p* = 0.001 for trend). **(C)** Comparisons of serum ALT levels between the patients with and without MAFLD (*p* < 0.001). **(D)** Comparisons of serum ALT levels across the serum iron quartile groups (*p* < 0.001 for trend).

The comparisons of serum ALT levels and the percentage of the patients with elevated ALT levels in different groups are displayed in **Figure 3**. After controlling for sex, age, and DD, serum ALT values and the percentage of the patients with elevated serum ALT levels were significantly greater in T2DM patients with MAFLD compared with those without MAFLD (*p* < 0.001, **Figures 3A, C**). Furthermore, both the percentage of the patients with elevated serum ALT levels (Q1: 1.90%; Q2: 4.40%; Q3: 6.00%; Q4: 11.70%; *p* = 0.001 for trend, **Figure 3B**) and serum ALT levels [Q1: 16 (11–25); Q2: 19 (14–29); Q3: 22 (16–36); Q4: 25 (17–37); *p* < 0.001 for trend, **Figure 3D**] rose with the increasing serum iron quartiles after adjusting for sex, age, and DD.

### Comparisons of HOMA2-IR and HOMA2-S

**Figure 4:**
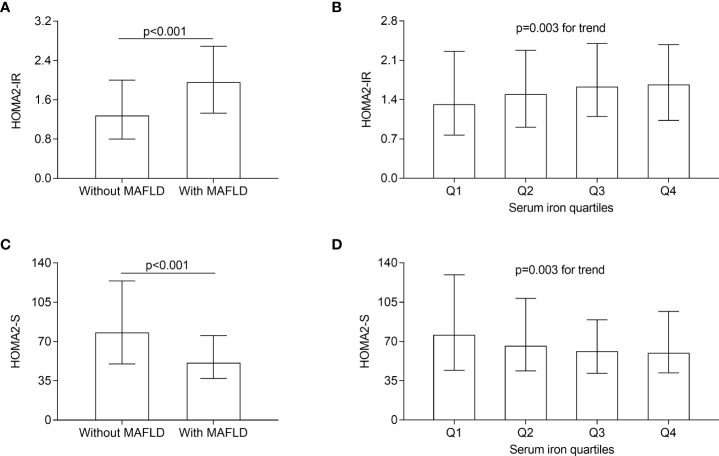
Comparisons of HOMA2-IR and HOMA2-S. **(A)** Comparisons of HOMA2-IR between the patients with and without MAFLD (*p* < 0.001). **(B)** Comparisons of HOMA2-IR across the serum iron quartile groups (*p* = 0.003 for trend). **(C)** Comparisons of HOMA2-S between the patients with and without MAFLD (*p* < 0.001). **(D)** Comparisons of HOMA2-S across the serum iron quartile groups (*p* = 0.003 for trend).

**Figure 4** illustrates the HOMA2-IR and HOMA2-S comparisons between T2DM patients with and without MAFLD as well as across serum iron quartile groups. After adjusting for sex, age, and DD, higher HOMA2-IR and lower HOMA2-S were observed in T2DM patients with MAFLD than in those without MAFLD (all *p* < 0.001, **Figures 4A, C**). Moreover, the significantly increased trend in HOMA2-IR (*p* = 0.003 for trend, **Figure 4B**) and the obviously decreased trend in HOMA2-S (*p* = 0.003 for trend, **Figure 4D**) were observed across the serum iron quartiles.

### Association of serum iron levels with MAFLD

**Table 2** shows binary logistic analysis for the association of serum iron levels with MAFLD in T2DM patients. Before (Model 1) and after adjustment for age, sex, smoking, alcohol use, DD, obesity, and hypertension (Model 2), the patients with higher serum iron levels showed an increased risk for MAFLD comorbidity (*p* < 0.001). Further correcting for the treatment of LLD, metformin, IIAs and insulin sensitizers (Model 3), physical examination data (Model 4), and laboratory parameters (Model 5), serum iron levels continued to be positively associated with the presence of MAFLD (all *p* < 0.001).

**Table 2:** Association of the prevalence of MAFLD with serum iron.

	*B* statistic	OR	95% CI	*p*-value
Model 1	0.311	1.365	1.223-1.523	<0.001
Model 2	0.329	1.390	1.224-1.578	<0.001
Model 3	0.313	1.368	1.202-1.556	<0.001
Model 4	0.345	1.412	1.220-1.633	<0.001
Model 5	0.545	1.725	1.427-2.085	<0.001

Model 1: Unadjusted.

Model 2: Adjusted for age, sex, DD, smoking status, alcohol intake, obesity, and hypertension.

Model 3: Further adjustment for use of LLD, IIAs, metformin, and insulin sensitizers.

Model 4: Further adjustment for SBP, DBP, WC, WHR, and BMI.

Model 5: Further adjustment for TC, TG, HDL-C, LDL-C, eGFR, Cr, SUA, UAE, HbA1C, FCP, 2-h CP, HOMA2-IR, FPG, 2-h PPG, ferritin, and CRP.

### Association of serum iron quartiles with MAFLD

**Table 3** shows the association of serum iron quartiles with the presence of MAFLD in T2DM patients, which was analyzed by binary logistic regression. In unadjusted analysis, higher serum iron quartiles presented an obviously increased risk of MAFLD (*p* < 0.001 for trend) (Model 1). After further adjustment for age, sex, smoking, alcohol consumption, DD, obesity, and hypertension, higher serum iron quartiles remained related to a higher risk of MAFLD (*p* < 0.001 for trend) (Model 2). After further correction for variables regarding medication therapy (Model 3) and physical examination (Model 4), increased serum iron quartiles were stably correlated with the presence of MAFLD (all *p* < 0.001 for trend). Finally, after controlling for laboratory parameters, the correlation between serum iron quartiles and the presence of MAFLD stayed significantly positive (*p* < 0.001 for trend) (Model 5).

**Table 3:** Association of the prevalence of MAFLD with serum iron quartile groups.

	ORs (95% CI)				*p*-values for trend
	Q1	Q2	Q3	Q4	
Model 1	1	2.298 (1.687–3.131)	2.241 (1.646–3.051)	2.432 (1.787–3.310)	<0.001
Model 2	1	2.551 (1.805–3.607)	2.463 (1.740–3.487)	2.496 (1.745–3.570)	<0.001
Model 3	1	2.315 (1.627–3.295)	2.246 (1.577–3.199)	2.370 (1.651–3.402)	<0.001
Model 4	1	2.588 (1.736–3.856)	2.429 (1.640–3.596)	2.532 (1.684–3.807)	<0.001
Model 5	1	2.944 (1.808–4.794)	3.185 (1.961–5.170)	4.009 (2.375–6.766)	<0.001

Model 1: Unadjusted.

Model 2: Adjusted for age, sex, DD, smoking status, alcohol intake, obesity, and hypertension.

Model 3: Further adjustment for use of LLD, IIAs, metformin, and insulin sensitizers.

Model 4: Further adjustment for SBP, DBP, WC, WHR, and BMI.

Model 5: Further adjustment for TC, TG, HDL-C, LDL-C, eGFR, Cr, SUA, UAE, HbA1C, FCP, 2-h CP, HOMA2-IR, FPG, 2-h PPG, ferritin, and CRP.

## Discussion

The prevalence of MAFLD among T2DM patients was 41.2% in the present study, close to our previous findings of 39.4%–52.6% in T2DM, which was diagnosed based on NAFLD without alcohol users (9–11). Even though the criteria of NAFLD was replaced with MAFLD including patients with alcohol consumption in the current study, the difference of prevalence was comparatively insignificant. Therefore, it was suitable to choose MAFLD as the definition. Consistent with our previous study, the prevalence of MAFLD was higher in women, middle-aged patients, and patients with a short DD in this study (11). Notably, the median age of the enrolled patients was about 60 years old. Previous studies noted that women over the age of 60 had a higher prevalence of MAFLD than men, possibly influenced by menopause in women (30, 31). Moreover, the peak prevalence of MAFLD was between the ages of 18 and 50 in other studies, which supported our suggestion that middle-aged people were more likely to develop MAFLD compared with older people (32, 33). Possible reasons for a higher MAFLD prevalence in younger patients were as follows: middle-aged people were more susceptible to sedentary lifestyles, obesity, and stressful socioeconomic status, which increased MAFLD risk; elderly patients experienced an increased overall mortality partially caused by fatty liver; and the deceased patients were excluded from the MAFLD population (34, 35). Additionally, the negative association between DD and MAFLD prevalence was partly explained by several studies, in which obesity and insulin resistance were involved in the early stages of T2DM as risk factors for MAFLD (11, 36, 37). Alternatively, as the duration of diabetes increased, the duration of glucose-lowering medication was correspondingly longer, some of which were thought to have a therapeutic effect on MAFLD and might lead to a reduction in MAFLD (38). Moreover, serum iron levels were higher in men than in women, but the MAFLD prevalence was lower in men than in women, which might be explained by the fact that MAFLD was influenced by multiple factors in addition to gender and serum iron. Additionally, serum iron levels decreased with increasing age and DD, which corresponded with a decrease in MAFLD prevalence with higher age and longer DD.

Currently, the correlation between serum iron and MAFLD remains unclear in the general population. For example, a cross-sectional study showed that the serum iron levels and the NAFLD prevalence had a negative correlation (16). Conversely, a recent study based on obese patients found that the severe NAFLD group had higher serum iron levels than the mild or moderate groups (39). Other studies suggested an irrelevant association between serum iron and NAFLD staging and liver fat content (17, 18). Likewise, besides few relevant investigations, there were conflicting opinions on the relationship between iron status and MAFLD in T2DM patients. For example, a previous study exhibited a positive correlation between iron store and the degree of NAFLD in patients with coexisting T2DM and NAFLD (40), whereas another study supported no correlation between NAFLD and hepatic iron in T2DM subjects (22). Therefore, we conducted the present study investigating the serum iron levels and MAFLD correlation in patients with T2DM.

Notably, the present study demonstrated that there was a positive correlation between serum iron levels and the prevalence of MAFLD. The risk of MAFLD increased nearly 1.73-fold with each 1 SD increase in serum iron levels. Consistent with our results, a study comprising subjects with T2DM noted 20% of patients with steatosis in the low serum iron group compared with 78.9% of patients with steatosis in the high serum iron group (13). Similarly, patients with NAFLD had an average body iron of 1.6 g compared with 1.4 g without NAFLD in a clinical trial based on T2DM and prediabetes (41). Furthermore, a previous study supported that a 12-month glucose-lowering strategy for poorly controlled T2DM patients enabled the simultaneous reduction of serum ferritin from 223 μg/L to 121 μg/L, hepatic iron concentrations from 109.2 mg/100 mg to 89.7 mg/100 mg, and the prevalence of MAFLD from 80% to 25% (42), which indicates positive correlation between iron and MAFLD in T2DM patients. Additionally, the prevalence of MAFLD increased at least threefold when serum iron was greater than 10.6 μmol/L. Even though the prevalence of MAFLD was the highest in Q4, all other groups actually had higher prevalence of MAFLD compared with Q1 (serum iron < 10.6 μmol/L). It suggested that there might be a threshold value of serum iron, beyond which the risk of MAFLD increases significantly. Given that iron depletion ameliorated MAFLD and that iron deficiency was also associated with the increased risk of metabolic dysfunction (43, 44), it might be required to maintain serum iron in a suitable range for treatment and prevention of MAFLD.

Moreover, the present study also suggested increased liver enzymes with the increase of serum iron levels, which reflected the aggravation of hepatocyte damage. Therefore, rising serum iron might be closely related to the severity of liver damage caused by MAFLD in addition to the increased presence of MAFLD. Similar to our findings, several studies also displayed a positive association between serum or body iron and ALT levels in patients with or without T2DM (39, 41, 45). For example, the NHANES study including NAFLD subjects without T2DM indicated a 1.13-fold risk of elevated ALT levels with increasing deciles of serum iron concentration (45). Interestingly, after induction of iron depletion to near-iron deficiency, there was nearly half of the reduction in serum ALT levels in MAFLD patients with T2DM, without descent in non-MAFLD patients with T2DM treated equally (41). Therefore, a possibly more severe hepatocellular injury was related to elevated serum iron, which might suggest the progression of fatty liver.

The reason explaining the close correlation of serum iron with MAFLD may be attributed to insulin resistance induced by iron, which was a major mechanism in the MAFLD pathogenesis (3). Iron metabolism disorders aggravate oxidative damage to hepatocytes and thus lead to insulin resistance and subsequent compensatory hyperinsulinemia, which promote hepatic *de novo* lipogenesis and cholesterol synthesis as well as reduce free fatty acid catabolism by oxidation (3, 8, 44). Moreover, the significant relationship of high iron status with insulin resistance has been repeatedly confirmed in many studies (17, 39, 41). For example, HOMA-IR was positively linked to serum iron with a correlation coefficient of 0.189 in obese patients with or without T2DM (39).

A previous study underlined that in patients with obesity and metabolic syndrome, the increase of serum iron was accompanied by elevated insulin resistance evaluated by HOMA-IR (39). A subsequent study speculated that the elevated iron levels could impair the function of pancreatic β cells and cause systemic insulin resistance in T2DM subjects (46). Furthermore, previous studies also detected that iron removal improved both insulin sensitivity and β-cell function in patients comprising T2DM (17, 39, 41). Understandably, we also proved higher HOMA2-IR, FCP, 2h C-P, and lower HOMA2-S from the lowest to highest serum iron quartiles. Therefore, increased insulin resistance caused by high iron levels might contribute to the development and progression of MAFLD in T2DM.

Verified by other studies, we also observed the elevation of serum ferritin in T2DM patients with MAFLD compared to those without MAFLD (13, 47). Although serum ferritin levels increased with the rising of serum iron quartiles, serum ferritin was no longer an independent risk factor for MAFLD in the binary logistic regression analysis. A previous study supported the idea that serum ferritin levels failed to correlate with NAFLD after adjusting for multiple factors (48). The possible reason for inconsistency was that serum ferritin reflected the stored iron in cells, which might not be fully associated with iron that exerted function (49). Moreover, CRP levels decreased from lower to higher serum iron quartiles in the present study. Consistently, in a study based on a mouse model, MAFLD was exacerbated accompanied by elevated serum iron but reduced CRP levels (8). Another study indicated the lack of correlation between CRP and iron status in T2DM patients (50). Moreover, no appreciable difference in CRP between non-NAFLD and NAFLD in some cases might suggest multiple factors instead of merely inflammation influencing MAFLD (5, 51).

We faced some limitations during the current research. First, some cases with milder steatosis were probably missed by ultrasonography. Even so, there was a good concordance between ultrasound diagnosis and pathological diagnosis in NAFLD patients (52). Moreover, ultrasonography was recommended as the first-line imaging method by the clinical guidelines for MAFLD, which ensured accuracy and convenience of ultrasound diagnosis to detect steatosis (29, 35). Second, although various factors might influence insulin resistance, we adjusted as much factors as possible including DD, medication usage, lipids, and so on to reduce the impact on the results. Third, the present study was conducted in a specific population and further population expansion was needed to confirm the findings. Fourth, since this is a cross-sectional study, it is difficult to determine the causal relationship between iron and MAFLD, whereas we supposed that serum iron might be one of the causes of MAFLD based on previous studies (8, 39, 43). It has been suggested that iron overload aggravated hepatic insulin resistance (8, 39), while iron depletion therapy ameliorated NAFLD (43).

## Conclusions

In conclusion, serum iron levels are independently and positively associated with MAFLD in patients with T2DM. Serum iron levels could be a biomarker to evaluate the risk of MAFLD for better screening and prevention in T2DM patients.

## Data availability statement

The raw data supporting the conclusions of this article will be made available by the authors, without undue reservation.

## Ethics statement

This study was reviewed and approved by Ethics Committee of Shanghai Jiao Tong University Affiliated Sixth People’s Hospital. The patients/participants provided their written informed consent to participate in this study.

## Author contributions

L-XL and M-FL provided the hypothesis, designed the study, and revised the manuscript. J-WW, C-HJ, and J-FK made contributions to the acquisition, analysis, or interpretation of data. J-WW drafted the manuscript. Y-LM, J-XL, and Y-JW participated in the revision of the manuscript. All authors contributed to the article and approved the submitted version.

## Funding

This work was supported by grants from the National Key Research and Development Plan (2018YFC1314900 and 2018YFC1314905), the National Natural Science Foundation of China (81770813 and 82070866), the Translational Medicine National Key Science and Technology Infrastructure Open Project (TMSK-2021-116), the Exploratory Clinical Research Project of Shanghai Jiao Tong University Affiliated Sixth People’s Hospital (ynts202105), and the Shanghai Municipal Key Clinical Specialty.

## Conflict of interest

The authors declare that the research was conducted in the absence of any commercial or financial relationships that could be construed as a potential conflict of interest.

## Publisher’s note

All claims expressed in this article are solely those of the authors and do not necessarily represent those of their affiliated organizations, or those of the publisher, the editors and the reviewers. Any product that may be evaluated in this article, or claim that may be made by its manufacturer, is not guaranteed or endorsed by the publisher.

## Abbreviations

T2DM, type 2 diabetes mellitus; NAFLD, non-alcoholic fatty liver disease; MAFLD, metabolic dysfunction fatty liver disease; DD, diabetes duration; SBP, systolic blood pressure; DBP, diastolic blood pressure; WC, waist circumference; LLD, lipid-lowering drugs; BMI, body mass index; HbA1C, glycated hemoglobin A1c; IIAs, insulin or insulin analogue; WHR, waist-to-hip ratio; FPG, fasting plasma glucose; 2-h PPG, 2-h postprandial plasma glucose; 2-h C-P, 2-h postprandial C-peptide; HOMA2-IR, HOMA of insulin resistance; TG, total triglycerides; TC, total cholesterol; FCP, fasting C-peptide; HDL-C, high-density lipoprotein cholesterol; LDL-C, low-density lipoprotein cholesterol; ALT, alanine transaminase; Cr, creatinine; SUA, serum uric acid; UAE, urinary albumin excretion; eGFR, estimated glomerular filtration rate; CRP, C-reactive protein.
